# Cancer Bioenergetics and Tumor Microenvironments—Enhancing Chemotherapeutics and Targeting Resistant Niches through Nanosystems

**DOI:** 10.3390/cancers15153836

**Published:** 2023-07-28

**Authors:** Aisha Farhana, Abdullah Alsrhani, Yusuf Saleem Khan, Zafar Rasheed

**Affiliations:** 1 Department of Clinical Laboratory Sciences, College of Applied Medical Sciences, Jouf University, Sakaka 72388, Aljouf, Saudi Arabia; 2 Department of Anatomy, College of Medicine, Jouf University, Sakaka 72388, Aljouf, Saudi Arabia; 3 Department of Pathology, College of Medicine, Qassim University, P.O. Box 6655, Buraidah 51452, Qassim, Saudi Arabia

**Keywords:** cancer chemotherapy, nanoparticles, metabolic regulation, bioenergetics, metabolic reprogramming, mitochondria, tumor microenvironment, theranostics

## Abstract

**Simple Summary:**

Cancer remains a major killer of the human population. Current cancer diagnostic and therapeutic methods are associated with shortcomings of limited targetability, specificity, solubility, and side effects. The therapeutic impediment is mainly attributed to the complex tumor microenvironments, which facilitate cancer progression. Additionally, the significant driver of tumorigenesis is mitochondria-centered energy metabolism. Bioenergetic alterations modulate the tumor microenvironment to help tumor progression and metastasis. In this review, we revisit the current understanding of mitochondrial bioenergetics mechanisms and tumor microenvironments that can be targeted through various nanoparticle-based smart systems. The nanosystems have gained momentum due to their high targetability and lower toxicity, and hence hold great potential in enhancing cancer chemotherapeutics. A liaison between nanoparticles and chemotherapeutic drugs can potentially target resistant cancers effectively for a successful therapeutic regime.

**Abstract:**

Cancer is an impending bottleneck in the advanced scientific workflow to achieve diagnostic, prognostic, and therapeutic success. Most cancers are refractory to conventional diagnostic and chemotherapeutics due to their limited targetability, specificity, solubility, and side effects. The inherent ability of each cancer to evolve through various genetic and epigenetic transformations and metabolic reprogramming underlies therapeutic limitations. Though tumor microenvironments (TMEs) are quite well understood in some cancers, each microenvironment differs from the other in internal perturbations and metabolic skew thereby impeding the development of appropriate diagnostics, drugs, vaccines, and therapies. Cancer associated bioenergetics modulations regulate TME, angiogenesis, immune evasion, generation of resistant niches and tumor progression, and a thorough understanding is crucial to the development of metabolic therapies. However, this remains a missing element in cancer theranostics, necessitating the development of modalities that can be adapted for targetability, diagnostics and therapeutics. In this challenging scenario, nanomaterials are modular platforms for understanding TME and achieving successful theranostics. Several nanoscale particles have been successfully researched in animal models, quite a few have reached clinical trials, and some have achieved clinical success. Nanoparticles exhibit an intrinsic capability to interact with diverse biomolecules and modulate their functions. Furthermore, nanoparticles can be functionalized with receptors, modulators, and drugs to facilitate specific targeting with reduced toxicity. This review discusses the current understanding of different theranostic nanosystems, their synthesis, functionalization, and targetability for therapeutic modulation of bioenergetics, and metabolic reprogramming of the cancer microenvironment. We highlight the potential of nanosystems for enhanced chemotherapeutic success emphasizing the questions that remain unanswered.

## 1. Introduction

Despite the advent of novel treatment modalities, chemotherapy remains the therapeutic backbone against all cancers; however, it is limited by diverse responses among various cancers and patients. Cellular mechanisms that regulate the proliferation of cancer cells and fabricate the overall tumor microenvironment are inherent to the sensitivity and success of chemotherapeutic modalities. Preclinical cancer models demonstrate a distinctive role of the cellular bioenergetics to modulate the immune responses within the TME and hence the chemotherapeutic outcomes [1,2,3].

Tumors reprogram their microenvironment primarily through metabolic alterations to achieve the biosynthetic and bioenergetic demands of increased proliferation and cell survival [4]. These metabolic changes are a hallmark of cancer progression, promoting unrestricted proliferation and metastasis and increased resistance of tumor cells to innate immune mechanisms and therapeutic agents [5,6]. Most cancer therapies are designed to induce cellular apoptosis or to skew or abrogate immune response [7]. Some of the key molecular players that regulate cancer progression underlie cellular bioenergetics. Mitochondria play a significant role in skewing the bioenergetics and apoptotic responses and are considered central for inducing or reducing cancer progression [8,9]. Hence, a thorough understanding of TME and underlying cellular bioenergetics specific to cancer development, progression, and metastasis will positively impact the development of novel therapeutic modalities.

A significant breakthrough in cancer chemotherapeutics is achieved through the use of nanoparticles. Nanoparticles with unique properties ranging in size between 10 and 1000 nm are among the most effective nanotechnology platforms [10,11]. Nanoparticles have gained significant traction in medicine due to their ability to deliver drugs and bioactive components directly to target cells and tissues at a specific rate [12,13]. The advent of nanoparticles has opened novel avenues for diagnosing and treating a wide variety of diseases [14,15]. There is no doubt that the use of nanoparticles in cancer theranostics has accelerated the development of novel diagnostic methodologies and therapeutic platforms and aided conventional cancer treatment and diagnostic methodologies. Integrating nanosystems into chemotherapy, radiation therapy, and surgery has minimized limitations such as poor selectivity, solubility, distribution, specificity, and adverse side effects [16,17]. Nanomaterials have enhanced the potential of advanced therapeutic methods such as photodynamic and photothermal therapies, tumor catalytic therapies, etc. [18,19,20]. Nonetheless, the full potential has not been achieved due to limited knowledge of TME and hence, the development of TME-targeted monotherapies. Tailoring nanomaterials toward metabolic targeting to modulate autophagy, aerobic respiration, glycolysis, immune mechanisms, resistant niches, and apoptosis may enhance conventional treatment methods. Smart nanomaterials can also be developed to understand the underlying biochemistry of tumors [21]. Nanotherapeutics that skew TME toward normal metabolism may significantly enhance and improve cancer management, diagnosis, and treatment. The central focus of this review rivets on all the major players involved in cancer progression encompassing metabolic plasticity, immune mechanisms, and resistant niches, which are regulated through intricate bioenergetics modulations. Finally, the potential of disrupting tumor-driven bioenergetics and tumor-TME interaction is evaluated in light of emerging nano-based therapeutics.

## 2. Tumor Microenvironment-Insights and Targetable Niches

Cancer evolves and progresses through a dynamic interaction of tumor cells and the supporting milieu to create an ambient TME for the growth and proliferation of oncogenic cells [22]. Constituting the non-cancerous milieu, TME presents a heterogeneous environment of stromal cells and extracellular matrix (ECM). The cellular component includes the immune cells, signaling molecules, stromal cells, fibroblasts, and blood vessels, while the ECM comprises collagen, elastin, fibronectin, and laminin. The synergistic relationship between the tumor and its microenvironment generates clonal evolution and heterogeneity, builds multidrug resistance, and immune evasion leading to cancer progression and metastasis.

### 2.1. Tumor Microenvironment

**Figure 1:**
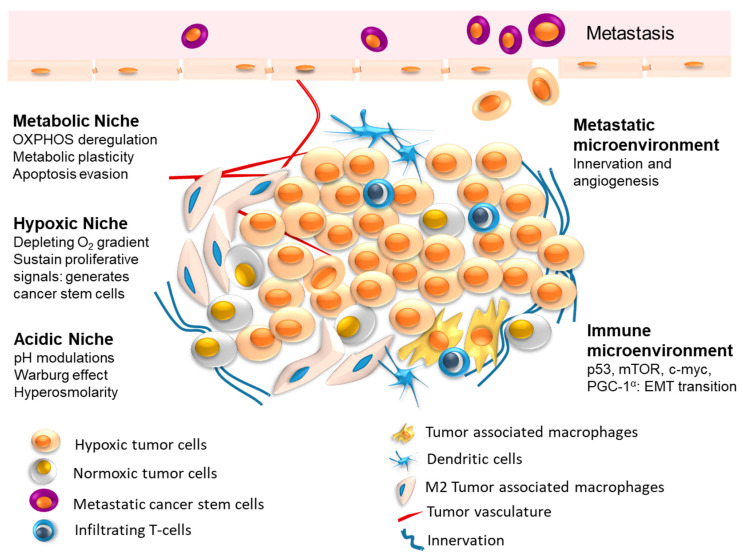
Tumor microenvironment landscape and targetable niches: Tumors are highly heterogeneous in the cellular and non-cellular milieu. The interplay among the tumor microenvironment components and crosstalk with the tumor and non-tumor components facilitates tumor growth and metastasis. The major tumor niches that modulate tumor infiltration and growth are depicted by metabolic, hypoxic, and acidic niches that create a distinct metastatic and immune microenvironment.

The TME is a highly integrated system, harboring several distinct specialized niches. Comprised of a plethora of resident and infiltrating host cells, soluble factors, ECM, and immune effectors, TME supports cancerous growth. The strength and extent of interaction between the tumor and the non-tumor cellular and non-cellular microenvironment play a decisive role in tumor initiation, development, and progression. The ability of tumor cells to invade and hijack non-malignant cells and disarm them of their function through cellular, molecular, and physical alterations leads to cancer proliferation within the surrounding milieu [23]. TME continuously evolves by generating intricate networks that are vary considerably among various tumor types [24]. TME also reprograms angiogenesis to restore oxygenation, nutrient acquisition, and waste elimination during intense growth [25]. Inherently, TME is also supported by vascular and lymphatic systems providing a supply and support mechanism for growing cancer at all stages of development and malignant transformation (Figure 1). Thus, tumor-derived components indigenous to tumor types and subtypes can be harnessed as effective cancer diagnostic modalities to precisely follow therapeutic success. The major components of TME are discussed below:

#### 2.1.1. Immune Component—Response and Dynamics

TME reflects a dichotomy within its immune components wherein some cells are protumorigenic while others are antitumorigenic [26]. Numerous innate and adaptive immune cells are a part of TME [27]. The adaptive immune mechanisms activate and enhance selective anti-tumor responses by following the signals triggered by tumor-associated antigens (TAAs), supported by immunological memory. The innate immune responses are activated upon TAA exposure and are executed by macrophages, monocytes, neutrophils, and natural killer (NK) cells. Despite TAA which triggers a host-induced immune response, tumors persist and proliferate by mechanisms that enable them to evade host immunity [28]. Tumor heterogeneity and continuous TME reprogramming also cause immunophenotypic modifications and, thus, resistance to immune mechanisms as well as chemotherapeutics [29]. Tumor-associated macrophages (TAMs), usually macrophage type 2 (M2) macrophages, are the key feature of the TME that facilitate tumor progression, invasion, angiogenesis, and metastasis. The M2 macrophages identified by surface expression of matrix metalloproteases (MMPs), Macrophage colony-stimulating factor (M-CSF), CSF-1 receptor (CSF1R), Programmed death-ligand 1/ligand 2 (PD-L1/L2), interleukin 10 (IL-10), prostaglandin, Transforming growth factor beta (TGFβ) are crucial in remodeling tumor tissue, subduing host immune responses channeling oncogenic microRNA (miRNA) loaded extracellular vesicles [30,31]. Tumor-destructive macrophage machinery is hijacked and remodeled through miRNA [32]. TAM control aerobic glycolysis and secrete various chemokines that propel metastatic transformation, invasive potential of tumors, promote epithelial-to-mesenchymal (EMT) transition, and intensify stemness [33]. Furthermore, TME is dotted with cluster of differentiation (CD)8^+^ T-cells, CD4^+^ T-cells, and FOXP3^+^ regulatory cells that circulate the microenvironment in variable percentages and are also observed in the peripheral blood in several cancers [34]. An interplay between CD8^+^ T-cells and CD4^+^ T-cells influences the clinical outcome.

Secondly, tumors employ upregulation of immune checkpoint inhibitory proteins (CIPs) to generate self-tolerance. The CIP bind with their partner proteins in a receptor-ligand interaction, facilitated through co-stimulatory molecules like CD80, CD86, CD40, inducible costimulator-ligand (ICOSL), etc., expressed by antigen-presenting cells (APCs) and T-cells [35]. Additional co-inhibitory signals such as cytotoxic T-lymphocyte–associated antigen 4 (CTLA4), Lymphocyte Activating 3 (LAG3), CSF1R, and PD1 further suppress T-cell activation, and act as modular mediators for tumors to exert immune evasion. The most commonly recognized co-stimulatory receptors on tumor-infiltrating lymphocytes (TILs) are the Tumor Necrosis Factor Receptor Superfamily (TNFRSF) and Immunoglobulin Receptor Superfamily (IgSF) receptors [36]. The upregulation of co-stimulatory receptors is mediated by T-cell receptor (TCR) engagement, however, the co-stimulatory receptors CD27 and CD28 are represented by their constitutive expression on the T-cell population. Both CD27 and CD28 are inherent in facilitating tumor progression [37,38].

#### 2.1.2. Stromal Cells and ECM Component

TME presents a critical intersection exerting beneficial and harmful effects depending on the cancer stage [39]. Within the TME, the cellular matrix is pivotal in preventing immune attacks and therapeutic challenges in both early and advanced tumor phases [40]. On the other hand, tumor cells also generate and control complex signaling networks to modulate the cellular and ECM components promoting tumor development, maintenance, and proliferation [41]. A striking result of this regulatory control is the development of multi-drug resistance (MDR) and abrogation of response to therapy [42].

The non-cancerous cells, such as cancer-associated fibroblasts (CAFs), endothelial cells (ECs), and mesenchymal stem cells (MSCs), are supportive matrix components that promote the developmental and metastasic phases [41]. Intercellular communication within the TME is mediated by a complex network of growth factors, chemokines, cytokines, and inflammatory and ECM remodeling proteins. Newer mechanisms of cellular interaction that promote horizontal gene transfer are currently emerging [43]. Circulating cancer cells, cell-free DNA, apoptotic bodies, and tumor-derived exosomes functioning to facilitate information delivery to other tumor or normal target cells are notable in cancer progression [44]. The CAF circumventing the cancer cells demonstrate immense heterogeneity and plasticity pivotal in providing physiochemical support for tumorigenesis by secreting ECM, inhibiting apoptosis, and facilitating the proliferation and migration of tumor cells [45,46]. TME resident CAF show enhanced cellular migration, angiogenesis, inflammatory cytokine signaling, and protumor metabolic adjustments. CAF also optimally maintains oxidative stress and nutrient flux to mediate mitochondrial autophagy and biogenesis in the surrounding cancer cells [47].

TME has a high number of tumor endothelial cells (TECs) and pericytes. TEC originates from the differentiation of the cancer cells or the EC that migrate within the TME. TEC provides sustainability and chemotherapeutic resistance by their stem cell-like behavior and overexpression of MDR1, aldehyde dehydrogenase (ALDH), etc. Studies have observed that TEC can develop self-renewal capacity and stem-like properties through nitric oxide-mediated notch signaling [48]. Pericytes (PCs) or mural cells envelop blood vessels and regulate vascular permeability, stability, and blood flow, serving as gatekeepers to tumor spread. Pericytes structurally support the endothelial cells enveloping them with extended cytoplasmic processes [49,50]. Involved in the development of vasculature, pericytes facilitate angiogenesis and hence contribute to tumorigenesis. Intriguingly, vascular pericytes under the effect of TGF-β facilitate the restructuring of tumor niche for supporting the vasculature [51]. They pose an inadvertent potential of tumor homing and effectively contribute to several hallmarks of cancerous growth.

A prominent component of TME, circulating tumor cells (CTCs) mirror the primary and metastatic tumors as they detach from the tumor site and enter the systemic circulation [52]. CTCs are one of the most potent diagnostic, prognostic, and sensing clinical tools that can signal the presence of undetectable tumors. CTCs are home to the tumor site through IL-6 and IL-8 cytokines secretion, and infiltration is mediated through MMP1, collagenase-1, and fascin-1 [53]. The maneuvering of CTC through systemic circulation facilitates seeding in other tissues and organs. Interestingly, this process is not unidirectional, and self-seeding in the original tissue leads to more aggressive phenotypes [54]. Prognostically, CTC can induce the recurrence of ablated tumors by self-seeding and stromal recruitment, which induce chemokines, vascularity, anaplasia, and an increase in tumor size. In dormant tumors, CTC can cause relapse, metastatic outgrowth, and compromise chemotherapy through drug resistance, posing a logistic, prognostic, and diagnostic threat [55]. Except for a few studies, a decline in CTC positivity reflects effective chemotherapy [56,57]. Chemotherapeutic regimes that target metastasis, including neoadjuvant or adjuvant, or combination therapies, have been largely effective in reducing CTC and the subsequent incidences of disease progression [58]. Despite the ambiguity associated with the diagnostic and prognostic value and applicability of CTC, they are a beneficial resource for therapeutic efficacy and potential for use in personalized medicine.

Exosomes secreted by tumor cells play a significant role in organ-targeted metastasis. Brain, lung, and liver-specific exosomes preferentially home to lung, epithelial cells, fibroblast cells, Kupffer cells, and brain endothelial cells [59,60]. The uptake of exosomes to the new niches initiates pre-metastatic priming of the tissue. The differential integrin expression that subsequently upregulates Src phosphorylation and S100 pro-inflammatory gene expression is essential in exosome-mediated interactions [61]. Exosomes carry the genetic and molecular signature of the originating cell and facilitate the transfer of tumorigenic, metastatic, and drug-resistance characteristics to other tumorous or healthy cells [62]. Like exosomes, apoptotic bodies released in the TME potentially regulate cancers associated immunity [33,63]. Apoptotic bodies package cellular contents spanning proteins, lipids, and nucleic acids (DNA, mRNAs, miRNAs) from the dying cells. Bystander cells internalize these cells to induce molecular memory and intracellular communication [64]. The horizontal transfer of genetic material through apoptotic bodies is instrumental in generating genomic diversity and enhancing the metastatic potential of tumors [65].

Sustained metabolic stress within the TME increases hypoxia and subsequent nutrient depletion. The resultant low oxygen and nutrient starvation induces AMP-activated protein kinase (AMPK), which inhibits the anabolic process and promotes the development of cancer-derived stem cells (CSCs), also termed as cancer-initiating cells (CICs) [66,67]. These CSC are a highly resistant, metabolically evolved cancer cell population generated through the EMT process progressing through the proliferative, epithelial (E), quiescent, and mesenchymal (M) and transition (T) stages [68]. A difference in the redox potentials and tumor heterogeneity distinctively characterizes each transition stage [69]. Two pathways, hypoxia-inducible factor-1-alpha (HIF1α) and nuclear factor erythroid 2–related factor-2 (NRF2), play an important role in regulating CSC transition during metabolic and oxidative stress [70]. Numerous studies have demonstrated that CSC preferentially exploits glycolytic metabolism instead of mitochondrial oxidative phosphorylation (OXPHOS) [71,72,73]. Though a dependence of CSC on glycolysis is frequently observed, some studies also demonstrate the reliance of CSC on oxidative metabolism [74]. It can be conjectured that precise targeting of CSC, enabled through both metabolic states, can potentially enhance the therapeutic efficacy of chemotherapy, radiotherapy, and antiangiogenic agents [75,76].

#### 2.1.3. TME Associated Resistant Niches

The tumor microenvironment comprises many specialized sections or niches that propel cancer progression through adaptation and survival processes [77]. These specialized heterogeneous anatomical compartments are integrated to generate an immune evasive and drug resistant environment [78]. Due to its diverse niches, each tumor displays different proliferation, progression, and metastasis (Figure 1). The recalcitrant nature of some of these niches has dampened most advanced cancer treatment modalities. A prominent cause of therapeutic failure is attributed to resistance to chemotherapeutic drugs, subsequently leading to recurrence and metastasis. Emerging studies elucidate that CSC or CIS are the major mechanisms of chemotherapeutic resistance in cancers, including colorectal, breast, bone, brain, etc. [79,80,81]. The CSC resides within a distinct microenvironments within the tumor, the CSC niche [82]. With self-renewal and repopulation capacity, CSC can initiate intra-tumor heterogeneity and diversification of cancer cell lineages. Hence, an inherent therapeutic resistance potential marks CSC as one of the most recalcitrant and refractory tumor niches [82].

Being the major driver of tumor heterogeneity, CSC facilitated therapeutic resistance limits overall patient survival. These stem cell niches induce specific gene expression events to support embryonic and stem cell development which reflecting high resistance to stressors such as hypoxia, antioxidants, and DNA damage responses [78,82]. Subsequently, the enhanced stress responses support therapy resistance, tumor plasticity, immune evasion, cancer proliferation, and metastasis [76]. Thus, understanding the compounded heterogeneity, regulatory networks and susceptibility of CSC niche is a pre-requisite to devise successful therapeutic strategies [82].

Recent advances in research have also highlighted the role of nutritional niches in regulating and overcoming therapy-induced senescence (TIS) [83]. Evasion of a senescent state is usually achieved through a non-proliferative form maintained with a basal metabolism. However, a long-standing TIS increases the propensity of tumor relapse and manifestation of the adverse effects of therapy. TIS cells persisting post-cancer therapy demonstrate high immune evasion [84,85]. These cells reprogram their metabolism into stem-like states with drug-resistance capabilities, ultimately contributing to cancer relapse [86,87]. TIS accumulation in inflammatory sites promotes EMT phenotypes that support metastasis. Like CSC, TIS can also induce epigenetically distinct senescent cell subtypes under nutritional and oxygen limitations [72,88,89]. Similarly, hypoxic niches regulate diverse facets of cancer development, including cancer cell epigenomic and epi-transcriptomic modulations, and cell-cell communication, etc. Intratumoral hypoxia shapes and facilitates the development of CSC [90]. Drugs that target intratumoral hypoxia have been developed but reflect clinic limitations of low targetability and relapse [91]. Molecular mechanisms through which hypoxia reshapes tumors and TME and adaptive responses can provide insight into developing hypoxic targeted tumor treatment. The primary driving mechanism that facilitates the development and stability of these niches is the bioenergetics modulation and resilience regulated through mitochondria [92].

### 2.2. Bioenergetics of TME and Tumor Logistics

#### 2.2.1. Mitochondrial Mechanics in Tumor Development and Proliferation

Mitochondria are integral to the metabolic and bioenergetics mechanisms required for cellular growth, proliferation, and survival. Vital cellular processes include energy metabolism, cellular redox (reduction–oxidation) status, and reactive oxygen species (ROS) and reactive nitrogen species (RNS) generation, innate immune mechanisms, regulation of cytosolic calcium levels, channelizing biosynthetic precursors such as pyrimidine and acetyl-CoA, and triggering apoptosis [93,94]. Being at the nexus of the metabolic junction, alterations in mitochondrial regulatory parameters result in deranged biosynthetic mechanisms, dysregulated cellular signaling events, and a switch from a quiescent cellular stage to a differentiating and subsequently highly proliferating state [93,95]. The normal physiology of mitochondria mitigates the abnormal cellular perturbations by regulating apoptosis through activating of mitochondrial permeability transition pore (mtPTP).

Mitochondria lie at the center of cancerous cellular transformations. Though extensive study, a lacuna exists in understanding the intricate crosstalk between mitochondrial mechanisms and cancer initiation and progression. Mitochondrial DNA (mtDNA) mutations that alter mitochondrial functions are evident in almost all cancers [96]. The high mutation rates of mtDNA create an admixture of normal and mutant mtDNA in the cells generating ‘heteroplasmy’ [97]. Increased mutant mtDNA content diminishes cellular energy output potential, resulting in an energy-deficient state. This bioenergetics stress experienced by mitochondria is mitigated through the fission and fusion process that complements damaged mtDNA [98]. Many cancers demonstrate aberrant fission and fusion mechanisms curtailing healthy cell growth and mitochondrial fragmentation. Emerging studies point out that the predominance of mitochondrial fission serves as a metastatic node in various cancers [99,100,101,102]. Highly fragmented mitochondrial pools can switch the cellular metabolic phenotype towards the formation of invadopodia and lamellipodia, increasing cellular motility, invasive potential, and, subsequently, metastasis [101].

Recent evidence demonstrates a marked correlation between mitochondrial shape and size with the manifestation of cancer [101,103,104]. Specific structural dynamics of mitochondria have been associated with cell cycle progression, metabolic adaptation, tumor growth, tumor cell motility, modulation of necroptosis, and autophagy [105,106,107]. Several fission-inducing proteins, like mitochondrial fission factor (MFF), Dynamin-related protein-1 (DRP1), and mitochondrial dynamics proteins-49 (MiD49), are often expressed more in cancer cells than healthy counterparts [108]. Studies have demonstrated that modulation of mitofusin (MFN1) and DRP1 drives the invasive and migratory potential of the cells [101]. Fission also serves as an important quality control for mitochondrial processes. Generalized fragmentation results in elevated ROS and loss of mitochondrial membrane potential, which activates specialized signaling pathways are activated to remove dysfunctional components through mitophagy [109,110]. The mitochondrial autophagic responses restrict ROS production and the release of apoptotic factors [111]. Studies have pointed out that tumor cells with defective mitochondria exhibit reduced tumorigenesis and metastatic potential. Such cells acquire mitochondria through cellular transfer to restore defective OXPHOS [112].

The adaptability of mitochondria impinges on cancerous transformation emanating from mitochondria, besides other factors. Hence, understanding mitochondrial mechanisms is critical to developing successful next-generation theranostic approaches for almost all cancers.

#### 2.2.2. Mitochondria Regulation of Redox Balance

A pivotal function of mitochondrial bioenergetics is the maintenance of redox homeostasis. Imbalances in the mitochondrial fission and fusion cycles are consequential to cellular and mitochondrial ROS generation and can lead to metastatic cellular phenotypes [101]. ROS plays a vital role in cancer biology as tumorigenic and tumor suppressive. The outcome underlies spatial, temporal, and concentration of ROS production and exposure. ROS is a significant modulator of signaling events, and a moderate increase in cellular ROS promotes tumorigenesis and metastasis [113]. Contrarily, large concentrations of ROS dictate cell death through apoptosis or necrosis [114]. The mitochondrial NADPH is central to ROS production and the reduction in thiols (-SH). Numerous protein functions are controlled by the reduction in –SH [115]. In line with cancer signaling, NADPH-Thioredoxin-1 (Trx-1) is instrumental in activating Apurinic/apyrimidinic endonuclease 1 (APE-1), which is a key regulator for various cancer signaling junctions such as nuclear factor-κB (NF-κB), NRF2, protein-53 (p53), Redox effector factor-1 (Ref1), estrogen receptor and glucocorticoid receptor [116,117].

#### 2.2.3. Mitochondria Mediates Integration of Environmental Cues into Cellular Bioenergetics

The cellular bioenergetics genes are dispersed across the nuclear chromosomes as well as mtDNA. The functional integration of these genes is regulated through cis and trans-regulatory networks that underlie mitochondrial signaling modules. Mitochondrial sensory mechanism integrate environmental cues into the adaptable cellular mechanism mostly through an epigenetic interface [118]. Key environmental modulators are the calorific input, wherein the energy funnels span the cellular glycolytic pathway and mitochondrial oxidative phosphorylation (OXPHOS), forming a circuit. The calories are converted to ATP, acetyl CoA, reduced NAD, and S-adenosyl-methionine (SAM). Energy abundance facilitates the replication and transcription by ATP and acetyl-CoA-mediated phosphorylation and acetylation of chromatin. On the other hand, calorific limitation causes dephosphorylation and deacetylation leading to the suppression of gene expression. Mitochondria also regulate SAM-mediated DNA methylation patterns pivotal to numerous signaling pathways through epigenetic events. Consistent with mitochondria-mediated epigenetic regulation, the clinical presentation of bioenergetics diseases simulates epigenetic pathologies, which also include cancer [119,120]. Thus, exploring the bioenergetics-epigenetic interface may also prove a logical approach to harness successful cancer treatment modalities.

### 2.3. Crosstalk between Mitochondrial Bioenergetics and TME-Driving Tumor Progression

**Figure 2:**
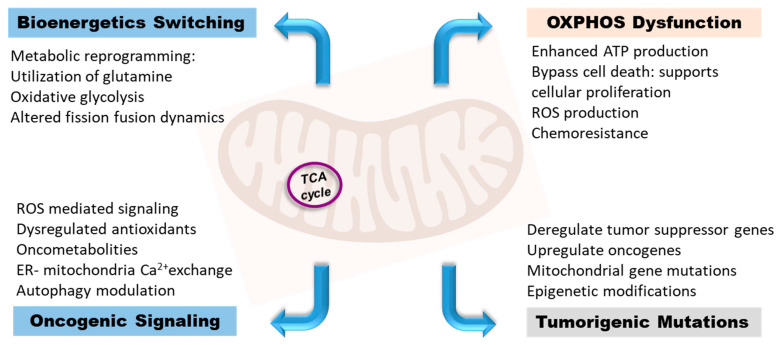
Mitochondria play a crucial role as decision makers of cancer progression. Inherent to their capabilities as a powerhouse of the cells, mitochondria control cellular biosynthesis, bioenergetics, calcium and redox homeostasis, biogenesis, and cell death. Mitochondrial function is ingrained in each stage of cancer progression spanning tumor initiation development, metastasis, and therapeutic responses.

Mitochondria are involved in the development and progression of cancers through five major mechanisms that mediate tumor metabolic reprogramming (Figure 2). Eukaryotic cells are metabolically maintained and replicate by regulating important mitochondria-centered cellular processes; cell proliferation, metabolic adaptation, Ca^2+^ homeostasis, and programmed cell death are dependent on mitochondrial function [121]. As an important mediator in the crosstalk between tumor cells and TME, mitochondria regulate energy channels that directly and indirectly affect tumor initiation, progression, and metastasis. The viability and growth of cancer cells depend on energy provided through mitochondrial bioenergetics modulations.

Metabolic reprogramming in response to environmental and cellular stresses within cancer and the TME is integral to metabolic resilience and bioenergetics adaptability. Notably, all cancers present with an adjustable metabolic dimension, wherein a competition to acquire metabolic resources exists between cancer and the surrounding milieu [122]. With the advent of advanced genomics and metabolomics, a remarkable plasticity of tumor metabolic and bioenergetics mechanisms have been uncovered, and several cancer-associated bioenergetics signatures have been identified [123,124,125]. Long-standing metabolic challenges can lead to cancer initiation, with subsequent demands as cancer progresses and metastasize. Metabolic stresses emanate during tumor progression, exerting a selective pressure to facilitate cancer evolution and the proliferation of the fittest clones [126]. The mitochondrial DNA reflects a high mutation rate, further increasing during cancerous growth. Cancers are associated with DNA mutations, mostly affecting the electron transport chain (ETC) subunits and TCA cycle [127,128,129]. Alterations in the genetic and epigenetic modules of the mitochondrial genome arbitrate the energy capacity, which declines with the accumulation of somatic mtDNA mutations. Hence, a bioenergetics view of the diseases underlies a unifying mechanism for metabolic diseases and cancers.

Excess lactate production in an oxygenated environment was considered a driver for tumor progression—The Warburg effect [129]. Biochemically identified as ‘aerobic glycolysis’, the Warburg effect is centered on mitochondrial dysfunction. Mutations spanning the genes that encode TCA enzymes lead to an increased accumulation of TCA cycle intermediates and promote tumorigenesis [130]. TCA enzymes fumarate dehydrogenase, succinate dehydrogenase, isocitrate dehydrogenase, citrate synthase, and aconitase were observed to be altered in cancer [131]. Mitochondrial abnormalities in the metabolic enzymes induce metabolic reprogramming to skew cells towards oxidative glycolysis to support increase in proliferative potential of tumor cells [131]. High lactate yielded by oxidative glycolysis creates an acidic microenvironment facilitating only the growth of acid-resistant phenotypes [132]. Hence, cancer cells achieve a distinctive growth advantage, intensifying their invasive properties and metastatic potential, thus debilitating the growth of surrounding cells.

Within the TME, cancer cells and CSC exhibit mitochondria-centered bioenergetics modulations [133]. They can functionally maneuver the available nutrients to generate reducing power, ATP, and metabolic substrates for an uninterrupted energy supply [134]. This empowers the cancer cells with a selective survival advantage within the harsh TME. The present challenge in cancer theranostics necessitates an in-depth understanding of tumor-driven bioenergetics switching mechanisms and flexibility that drives cancer initiation, progression, and metastasis. The long-standing notion that carcinogenesis is dictated by selective induction of tumor-promoting oncogenes and switching off tumor suppressor genes now warrants further insights. Recent studies have proven that bioenergetics and cancer stem cells play a redefining role [76,134,135]. It is evident that cancer genetics-epigenetics intercepts tumor bioenergetics to drive tumorigenesis.

#### 2.3.1. Altered Mitochondrial Energy Metabolism in Cancer

The central energy transformation mechanism inherent to mitochondria is OXPHOS, an energy channelizing mechanism that powers several functions. In healthy cells, disturbances in the energy production pathways are sensed by mitochondria to initiate cell death through the mitochondrial permeability transition pore (mtPTP). In cancers, such metabolic perturbations are overcome by utilizing alternate substrates. As an obvious indicator of metabolic flexibility, tumor cells use glutamine as an OXPHOS substrate in addition to glucose [136,137]. In many cancerous cells, a significant amount of the ATP need is fulfilled through glutamine, even when a high-concentration of glucose is available [138]. Fructose and galactose can also serve as a carbohydrate source; however, 98% of energy is derived from glutamine through aerobic oxidation. It is also demonstrated that glutamine increases oxygen consumption. Many non-small cell lung cancer (NSCLC) types substantiate aerobic respiration through glutamine without glucose [139]. Though glucose and glutamine represent the key nutrient components that support energy needs at various stages; glutamine is a preferred substrate for OXPHOS in cancers [140,141]. Varying glucose and glutamine dependence is observed in different cancer cells to synchronize the cellular energy need generating building blocks and reducing power [137].

Metabolic substrates provided by cellular components of TME are also used by cancer cells for energy production. Heme is an essential component that affects mitochondrial biogenesis and OXPHOS reaction. Heme flux is increased in NSCLC and ovarian cancers to modulate oxygen consumption for supporting tumor progression [142,143]. Experimental evidence also suggests that ATP production is more significantly affected by OXPHOS in cancer cells compared to healthy metabolic cells [144]. The metastatic and circulating cancer cells utilize OXPHOS to produce a large amount of ATP [73]. OXPHOS is a preferred energy pathway in CSC, which exhibit higher oxygen consumption rates, ROS production, and a substantial increase in mitochondrial mechanics compared to non-stem cancer cells [145]. Further, mitochondrial ROS also promotes Kirsten rat sarcoma viral oncogene homolog (KRAS)-induced anchorage-independent cancerous growth [146,147].

The role of the kynurenine pathway was recently identified in the development of various cancers. This pathway generates many immunosuppressive metabolites and NAD^+^ by the enzymatic conversion of tryptophan, contributing to the cellular NAD^+^ pool [148]. NAD^+^ activity is usually localized to mitochondrial metabolism in normal cells; however, it is channelized to foster high ATP demands and metabolic reprogramming in cancer cells. In proliferative cancers, reduced levels of tryptophan and elevated concentration of kynurenine (tryptophan metabolite) promote cancer development, as observed in many cancer types [149,150,151,152]. As well as providing reducing equivalents, kynurenine pathway metabolites subdue natural killer (NK) cell function by interfering with NKp46 and Natural Killer Group-2D (NKG2D) receptors, downregulating cytokines, dampen immune surveillance mechanisms to promote the immune escape of cancer cells [153].

#### 2.3.2. Mitochondrial Redox-Bioenergetics Liaison

Though a visible Warburg effect is evident in proliferating cancer cells, the metabolic phenotype of metastatic cells is highly distinct from proliferating cells [154]. The oxidative glycolysis (Warburg effect) facilitates cellular proliferation by rapidly generating ATP synchronized with pentose phosphate pathway’s (PPP) flux. This supports biosynthetic activities and redox balance; the cancer cells switch their regulatory phenotype during hypoxic growth through the ROS machinery. Hypoxia, within the TME, also causes the cells to engage in stress mitigation as a survival strategy [155]. These stress dissipation responses are mediated by oncogenes and signaling events that regulate aerobic glycolysis and ROS concentrations. As a hallmark of cancer, proto-oncogenes such as c-myc, p53, KRAS, Rb, and liver kinase B1 (LKB1) gene, protein kinase B (AKT), Mammalian target of rapamycin(mTOR), and hypoxia-inducible factors (HIFs) are often dysregulated in tumors [156]. The c-myc, transcription factor, and PI3K and AKT-mTORC1 pathways integrate bioenergetics mechanisms to redox regulation. The PI3K -AKT-mTORC1 induces glutamine importers within cancer cells, switching metabolism towards TCA and one carbon to synthesize nucleotides and heme for proliferating tumor cells [75,157,158]. Moreover, sublethal doses of hydrogen peroxide induce caveolin-1 (Cav-1), which potentiate cancer cells to resist anoikis and allows anchorage-independent growth through Akt signaling [159,160].

During the metastatic phase, the detachment of cells from ECM disrupts various metabolic processes to increase anoikis susceptibility [161]. As discussed in the previous section, the cells shift to utilizing glutamine, which favors dissemination and maintenance of the redox balance. Many reports show that detached cancer cells induce ROS detoxification and bioenergetics skewing for optimal ATP production in low nutrient conditions [96,162]. Moreover, mitochondrial redox parameters play a crucial role in modulating metabolic adaptation to bypass metabolic constraints at each phase of cancer transformation. Several well established oncogenic mutations also affect signaling pathways to modulate cancer cell metabolism and ROS production [113,163].

Glutathione is another antioxidant molecule that maintains NADP+/NADPH ratio and subsequently redox homeostasis. Its concentration in the mitochondrial matrix equals that of the cytoplasm. In cancers, an increased glutathione synthesis dissipates the accumulation of dangerous levels of ROS, thereby balancing the intricate antioxidant concentrations crucial for cell survival [164]. The conversion of glutamine to oxaloacetate in cancer cells reverts NADP^+^ to NADPH, which directly affects ETC-derived O_2_ and other ROS. NADPH indirectly facilitates the re-reduction in glutathione disulfide (GSSG) to reduced glutathione (GSH) by glutathione reductase [165]. The redox state of glutathione is instrumental in regulating the tumor-associated factors, such as HIF-1, which induces angiogenesis in the tumor environment [166]. Cyclically, bioenergetics pathways engendered by glutaminolysis promote ROS, which is maintained through the TCA cycle and intricate antioxidant systems, imparting survival and proliferative advantages for cancerous growth [128].

#### 2.3.3. Mitochondrial Bioenergetics Directs the Modulation of Immune Mechanisms

Within the cancerous microenvironment, the immune cells encounter harsh environments and nutrient competition with the tumor cells. Furthermore, tumor cell mitochondria reprogram their energy metabolism to downregulate an array of anti-tumor immune mechanisms [167]. In vivo and in vitro murine models have shown that glucose depletion and subsequent accumulation of lactic acid within the TME abrogates T-cells function [168]. Normally, T-cells dynamically shift their metabolic program to anabolic biomass accumulation upon antigen encounter. This increases ATP demand and shifts the T-cell to aerobic glycolysis through the uptake of glucose and amino acids. However, tumor-regulated bioenergetics events induce switching of T-cell anabolic profile to catabolism within the nutrient-deprived TME, which serves as a key tumorigenesis driver [169]. Glucose deprivation also restricts cytokine production and the function of tumor-infiltrating T-cells. Tumor-cells derived lactate blocks lactate export from T-cells, further disrupting their aerobic glycolysis. Under nutrient-deprived conditions, AMP-activated protein kinase (AMPK) increases the AMP: ATP ratio regulating ifn-γ gene translation [170]. Nutrition unavailability also suppresses TCR-dependent Ca^2+^ NFAT (nuclear factor of activated T cells) signaling, hence T-cell hyporesponsiveness against tumors [171,172]. With the abrogation of pro-inflammatory and anti-tumor IFN-γ response, tumor proliferation continues unabated. On the other hand, CD4^+^Treg cells subdue inflammation, promote tumorigenicity, and are associated with poor prognosis in cancer patients. These tumor-infiltrating CD4^+^Tregs modulate their energy metabolism towards OXPHOS and lipid oxidation to generate energy [173]. Studies from mouse models have shown that Treg cells express low GLUT-1 and are not dependent on glucose uptake or glycolysis [174].

Furthermore, B lymphocytes support T-cell function and sustain adaptive immunity by generating TAA-specific antibodies, thus playing a prominent role in anti-cancer immunity [175]. Activated B-cells are metabolically highly active, demonstrating glucose uptake and lactate generation. Interestingly, the role of TCA and OXPHOS has been shown during the activation of naïve B-cells [176,177]. Hence, the competition for nutrients between the tumor cells and B-cells is instrumental in defining the immunosuppressive feature of TME. Another factor that abrogates tumorigenesis is NK cells [169]. These cells activated through IL-15 induce mTOR signaling to induce favorable bioenergetics [178]. Additionally, transferrin receptor CD71 and chaperon CD98 (amino acid transporter) are expressed on their cell surface. This facilitates NK cell proliferation and cytolytic ability [179]. Studies have demonstrated that TME-associated NK cells reflect impaired glucose metabolism that abrogates their functions [180]. Neutrophils are another vital immune component that demonstrate functional diversification. They release proteases and increase angiogenesis to facilitate cancer cell invasion and dissemination. Neutrophils orchestrate the rewiring of anti-tumor immune mechanisms by enhancing ROS production that subsequently suppresses NK and T-cell activities [181]. A study established that the mitochondrial respiratory potential of TME and tumor-associated neutrophils is channelized to produce ROS [127]. In conditions of restricted nutrient availability and hence low NADPH, oxidative glycolysis in neutrophils supports tumor growth and ROS-mediated T-cells suppression [179]. Therefore, similar to the glycolytic-neutrophils in the healthy host environment, mitochondrial pathways in tumor-associated neutrophils can be modulated toward possible cancer therapeutics.

A link between the inner and outer mitochondrial membrane proteins and apoptosis is also integrated through mitochondrial bioenergetics modulation [92]. The hemeprotein cytochrome c, associated with the inner mitochondrial membrane (IMM), mediates electron transfer from the respiratory chain to complex III to complex IV. The movement of electrons regulates bioenergetics flow and cellular ATP generation. During cancer growth, cytochrome c collaborates with ubiquinol as an essential juncture in caspase-mediated apoptosis [182]. Furthermore, outer mitochondrial membrane (OMM) is also a site of phosphorylation for many signaling proteins that function through bioenergetics modulation. Perturbations in kinase activity lead to changes in electron transport and subsequently the downstream cellular events as diverse as metabolism, cell cycle regulations, ETC modulations, etc. [183,184]. Protein kinase A (PKA), which binds cAMP, is OMM resident. Elevated PKA levels cause the hyperphosphorylation of complex IV, affecting the overall electron flow [185]. Complex IV activity co-operates with several oncogenes to support tumorigenesis. The binding of cAMP to PKA results in the activation and subsequent phosphorylation of several splicing factors (SRSF1, SRSF2, and SRSF9), shown to promote tumorigenesis in many cancers such as TNBC, lung cancers, colorectal cancers, etc. [186,187,188].

Another important kinase circuit integrated into mitochondrial bioenergetics control and essential for cancer progression is the phosphoinositide 3-kinase (PI3K)/AKT (protein kinase B) transduction pathway. AKT can phosphorylate several elements of ATP-synthase to enhance ATP production with subsequent inactivation of pro-apoptotic proteins to induce cell survival [189]. PI3K pathway activation affects mitochondrial energy mechanism through Protein Kinase C (PKC). Activated PKC prevents mitochondrial injury during cellular stress [190]. Proto-oncogene tyrosine-protein kinase (SRC) is another protein that localizes to the mitochondria as a molecular switch to control mitochondrial function affecting glucose metabolism [191]. SRC kinases phosphorylate ETS, such as Complex I subunits, to elevate respiration. It also activates succinate dehydrogenase (Complex II) to adapt mitochondrial dynamics and fuels cancer cells under restricted nutrient availability [192].

The present understanding entails that clonal evolution, genetic heterogeneity, and epigenetic modulations underlie the bioenergetics versatility of cancer cells and the TME. The ability of the tumors to relapse and reflect chemotherapeutic resistance is also ingrained in the mitochondria-centered bioenergetics responses. Although cancer-associated gene mutations are observed only in a few metabolic enzymes, numerous mutations in transcription factors and signaling networks can induce changes in the metabolic enzyme activity or expression patterns. The role of TME-associated cells in remodeling cancer bioenergetics is now actively researched to gain theranostic insights. Hence, a better understanding of tumor bioenergetics in relation to the TME should provide a comprehensive chemotherapeutic approach.

### 2.4. Theranostic Insights in Tumor-TME-Bioenergtics Interface

A significant hallmark of tumorigenesis is represented by metabolic rewiring, and its interaction with the TME serves as a critical juncture of diagnostics and therapy [193]. Even though cancer theranostics have gained visible success, there is high variation in the patient response in almost all types of cancers. Advanced therapeutics have also failed to achieve the estimated success rates attributed to the wide gap in our understanding of TME and the role of bioenergetics in dampening the otherwise effective chemotherapeutics. Therefore, insights into the molecular and cellular mechanism that underlies cancer bioenergetics is inherent to developing effective treatment modalities.

Thus far, static metabolic profiling has been the basis for understanding cancer bioenergetics leading to the advent of metabolic therapies [194]. This was counterintuitive with the exquisite heterogeneity and ever-changing energy demands of proliferative tumors. Hence, a reason for the limited success of metabolic chemotherapies [195]. The inherent need to envisage tumor-driven metabolic plasticity entails achievable therapeutic success. Nonetheless, two approaches of metabolic cancer chemotherapy, namely, press-pulse intervention and pharmacological targeting, can be amalgamated with the knowledge of metabolic flexibility to enhance the therapeutic outcome [196,197]. Secondly, in vitro modular platforms and patient-based models are needed to mirror cancer bioenergetics. A recent breakthrough to overcome translational bottlenecks is the metabolic pathway assessment is the Seahorse bioenergetics measurements, a sample-based customizable approach [194,196]. In sum, metabolic chemotherapy presents a promising cancer management interface subjected to patient stratification and standardization methodology.

TME undergoes numerous molecular and cellular transitions due to overall bioenergetics alteration and nutrient availability. The energy alterations in. duce redox changes and trigger TME and tumor to change their phenotype aimed to mitigate oxidative stress. One of the mechanisms is the EMT that induces metastasis. The tumor cells gain motility and infiltrate the TME by the action of TGFβ and epidermal growth factor. Furthermore, bioenergetics modulation can also trigger drug resistance and stem-like features during the cancerous transition, observed in many gastrointestinal, colorectal, liver, and lung cancers [8,198,199,200,201]. Hence, TME, protumorigenic molecules and EMT signaling events are potent chemotherapeutic targets mandating a scoping insight into their bioenergetics nature that will enable a strong foothold for developing TME-based therapeutics [202] In combination with conventional chemotherapeutics they can be used to circumvent drug.

Mitochondria-driven bioenergetics alterations, which generate CSC through stress-induced evolution process, is the major bottleneck in chemotherapeutic success. The CSC’s selective advantage in generating ATP within the nutrient-deplete TME makes them refractory to various drugs. The CSC is highly plastic, self-renewing, pluripotent cells equipped with chemoresistance, invasive abilities, and relapse [203]. However, manipulating or targeting bioenergetics’ vulnerabilities will engender unprecedented potential to decelerate CSC-induced cancer chemotherapeutic resistance. Currently, several CSC inhibitors have shown success in treating refractory cancers in combination with immunotherapy [204]. Different stages of CSC (E and M) show metabolic and redox differences [203]. Hence, each stage demonstrates the difference in sensitivities to redox or glycolytic inhibitors [205]. Dissecting the metabolic rewiring tuned by the specific regulators can pave way to curb CSC plasticity in various cancers.

Furthermore, CSC can detach from the cancer site, move, and persist into the TME or systemic circulation as circulating tumor cells (CTCs). In many circulating tumor cells (CTC)-induced metastatic cancers, such as breast cancer, melanoma, and lung cancers, energy is channelized through the pentose phosphate pathway (PPP) [162,206,207]. The PPP generates glutathione as an antioxidant to survive oxidative stress effectively. These cells harbor specific signatures that precisely demarcate primary from metastatic tumors, suitable for cancer detection, diagnosis, and therapeutics [208]. Additionally, CTC-based metastasis is supported by cellular metabolic plasticity and phenotypic flexibility to adjust within the new TME [209,210]. Studies have noted an increase in serine, asparagine, and proline metabolic enzyme expression in the metastatic CTC [187]. Increased PPP activity and enhanced acetate, pyruvate, and lactate uptake in these cells provide alternate forms of energy to support metastasis initiation and establishment [2,188]. Overall, current knowledge affirms that bioenergetics-targeted strategies can be instrumental in expanding the repertoire of combinatorial therapeutic regimens in the current cancer treatment modalities.

Tumor-derived circulating materials like cell-free DNA (cfDNA) are among potential cancer diagnostic, prognostic, management, and follow-up surrogate markers. cfDNA shows a complex release pattern attributed to the interconnected cellular process and originating sources. Studies in eight different cancer cell lines and patients’ plasma samples have elucidated the characteristics of cfDNA, confirming its release exclusively by cancer cells, with different release patterns [211,212,213,214,215,216]. Screening of the bioenergetics flux parameters showed a correlation between cfDNA release patterns and cellular origin, cancer status, and proliferation phase [217,218,219,220]. A significant dependence correlating with cfDNA release was noted with aerobic glycolysis but not OXPHOS [219,221,222]. cfDNA fragments bear unique genetic and epigenetic patterns characteristic to the tumor they originate [222,223]. Understanding of their physical properties and circulation dynamics can facilitate cfDNA optimization and refinement to harness effective theranostic uses [224,225]. Furthermore, kinetic evaluation and molecular profiling through BEAMing (beads, emulsions, amplification, and magnetics), next-generation sequencing (NGS), and digital droplet PCR (ddPCR) are potential non-invasive cancer management modalities. Recently, a surge in cfDNA-based identification of pathological signatures, staging, and development of analytical methods for cancer theranostics with clinical translations has been observed [226,227]. Hence, the significance of cfDNA in cancer diagnosis and precision medicine is underscored by its potential and routine use in the clinical diagnosis and management of several cancers.

TME-associated molecular, cellular, stromal, and signaling mechanisms are restructured by mitochondrial energy modulations to facilitate growth and resistance to therapy [214,220]. Hence, TME-associated tumor vasculature, immune cells, and stromal cells recruited to the tumor in all development and proliferative phases can be manipulated to manage cancer by altering or halting tumor advancement [199]. Metabolic derangement within the TME leads to a complex increase in gasotransmitters (nitric oxide, carbon monoxide and hydrogen sulfide), and metabolites such as lactic acid, K^+^, and kynurenine [220,228,229]. These molecules alter the pH of TME in a resource-constraint environment, repress immune functions, and help tumor growth. Studies have shown that modulating the bioenergetics of the cellular milieu underscores chemotherapeutic success [230,231].

### 2.5. Metabolic Reverse Programming as a Therapeutic Stronghold

Cancer bioenergetics directed modalities can be pivotal to expand and enhance combinatorial chemotherapeutics regimes to reverse program cancer cells. Currently, clinically approved immune checkpoint blockers have emerged as therapeutic metabolic regulators, some of which are discussed here.

Low glucose or lactic acid in the TME results in increased expression of checkpoint inhibitors [232]. Inhibitors that block the expression of programmed cell death 1 ligand 1 (PD-L1), which otherwise increases glycolytic activity and competition for glucose with T-cells can skew cancer cell metabolism [123,232]. As a combinatorial approach, adding anti-PD-1 therapy before conventional chemotherapy can benefit immune active tumors. Additionally, glycolytic enzyme lactate dehydrogenase A (LDHA), which converts pyruvate to lactate, is associated with tumor initiation, development, and metastasis. With aberrantly high expression in almost all cancers, LDHA is an established prognostication marker, also associated with H_2_O_2_ production and drug resistance [233]. Recent preclinical and translational studies using LDH-inhibitors in combination with existing chemotherapeutics demonstrate synergistic targeting of oxygenated cells [234,235]. Specific targeting of metabolically distinct cell populations associated with tumors or TME can therefore be an excellent therapeutic possibility [236,237].

Though metabolic processes are apparently similar to both cancer and immune cells, bioenergetics plasticity and heterogeneity delineate the two cell types [5]. Specific metabolic vulnerabilities of immune cells, within the TME, compared to cancer cells provide a window for enhanced immunotherapeutic interventions. Modalities are being developed to improve the anti-tumor response to checkpoint blockades, such as agonistic monoclonal Ab targeting co-stimulatory receptors, multiple checkpoint blockers, chimeric Ag receptor (CAR) T-cells, and cytokines [35,238]. Bioenergetics-targeted immunotherapy through immune checkpoint inhibitors (ICIs) targeting PD-1, PD-L1, and CTLA-4 has also revolutionized cancer treatment [239]. Studies on bioenergetics of tumor-infiltrated lymphocytes (TILs) within the TME and the immune evasion mechanisms elucidated the concept of CD8 T-cell exhaustion, critical for improving cancer immunotherapy. T-cell exhaustion is linked to overexpression of immune checkpoint receptors, such as PD-1, CTLA-4, T-cell Ig and mucin-domain containing (TIM)-3, and lymphocyte-activation gene 3 [240,241].

Owing to the complex nature of tumor-TME-immune interactions, anchored in unique mitochondria-centered bioenergetics reprogramming, the response to ICI treatment substantially varies among patients and cancer types. Hence, there is a need for dynamic metabolic biomarkers instead of static ones to predict the therapeutic response accurately.

### 2.6. Nanosystem Based Theranostics-Targeting TME and Bioenergetics to Encounter Resitant Niches

Chemotherapeutic management of various stages of cancer depends on accurate diagnosis and insightful follow-up. Early recognition of site, type, and stage is an inherent feature of tumor diagnostics and therapeutic mitigation of various cancers. Diagnostic modalities impinge on identifying tumor-associated molecular alterations through targeted chemical agents, drug trackers, imaging biomarkers, pH perturbation-responsive diagnostics, etc. [122,242,243,244]. Secondly, tracking therapeutic drugs within the systemic circulation, TME, and ultimately to the tumor site provides molecular insights into understanding the kinetics of drugs and the fate of chemotherapy [245,246]. The recent modular interface that has provided promising results in many clinical trials is the integration of nanosystems in cancer chemotherapy and diagnostics, further enhanced through the incorporation of smart nanomaterial [247,248,249,250]. Small molecule inhibitors, prodrugs, natural compound derivatives, and repurposed or synthetic drugs that demonstrated toxicity due to non-specific interactions were enabled to overcome drug resistance through novel nanosystems [248,251,252,253]. Nanosystems encapsulated or functionalized with chemotherapeutic drugs or as diagnostic tools have hugely augmented chemotheranostics toward a safe and successful clinical transformation.

#### Targeting Tumor Bioenergetics and Resistant Niches through Nanosystems

The cancer cells and TME exhibit resistance towards many structurally and functionally different chemotherapeutic drugs attributed to the dynamic, multifactorial events within the resistant niches. Mechanistic events, i.e., sustained proliferative signals, evading immune mechanism, apoptosis, and growth suppressor signals, maintaining replicative immortality, facilitating invasion and metastasis, are some of the drug refractory hallmarks of cancer [254]. Effectively controlling these dynamic events is centered upon energy metabolism alterations [25]. As discussed in earlier sections, the major player in maintaining the drug refractory potential of these niches is the metabolically diverse CSC, which harbors a distinct ability of bioenergetics modulation to switch between dormant and active phenotypes [255]. Notably, cancer-associated bioenergetics signatures are one of the most promising targets for nanomaterial-based therapeutic intervention.

**Figure 3:**
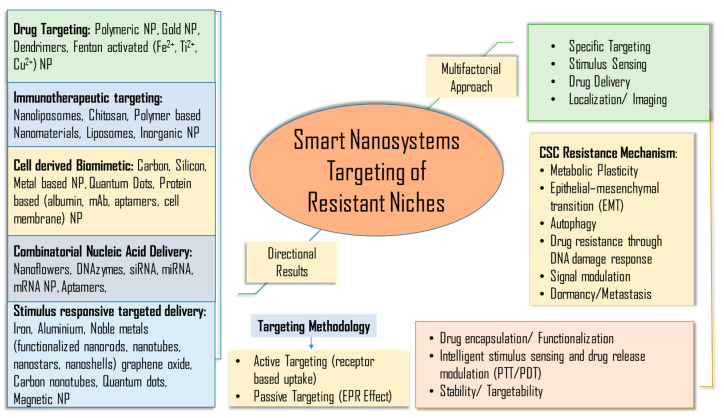
A schematic representation of smart nanosystem to target resistant cancer niches, especially the CSC niche. The flow diagram integrates the multifactorial approach using Nanoparticles (NP) for pan-cancer theranostics, aimed to obtain specific targeting.

Research focus has now shifted to enhancing bioenergetics targeting of cancers with using advanced nanoplatforms—smart nanosystems (Figure 3) [256]. Many studies have documented the ability of a multipronged approach with a rationally designed drug nanoconjugate to restore cellular OXPHOS or induce therapeutic bioenergetics changes. Some examples include multifunctional synergistic delivery, site-specific delivery, co-drug delivery nanosystems, etc. [257,258,259,260]. Studies have also demonstrated nanoencapsulation targeted therapeutics to bypass MDR mechanisms associated with TME and CSC, and have also shown it to modulate metabolic reprogramming repair, and DNA repair pathways within the cancer cells and TME [261,262,263,264,265,266,267]. The smart nanomaterial repertoire proven successful in overcoming chemoresistance spans polymeric frameworks, dendrimers and noble metals, organic systems, aptamer, hybrid nanomaterials, etc. capable of functionalization or encapsulating cancer-specific inhibitors, monoclonal antibodies, drugs, peptides, etc. [268,269,270,271,272]. (Figure 3).

CSC overexpress ABC drug efflux pumps such as P-glycoprotein (P-gp; ABCB1; MDR1), MRP-1 (ABCC1), and ABCG2 after chemotherapy initiation [273]. These transporters channel bioenergetics to fuel their function of reducing the bioavailability of chemotherapeutic drugs [274,275,276]. ABC transporters are inherent to absorption, distribution, metabolism, excretion, and toxicity (ADMET) of chemotherapeutic drugs [274]. The first-generation ABC transporter (P-gp) inhibitors that were effective in-vitro demonstrated pharmacological limitations. Likewise, second and third-generation ABC transporter inhibitors also caused interference with chemotherapy causing pharmacokinetics disturbance and cytotoxic side effects [277,278,279]. Fourth-generation ABC efflux pump inhibitors are natural compounds and flavonoids that demonstrated therapeutic efficacy with no toxicity [280]. However, in spite potent inhibition, therapeutic success was limited due to low solubility and bioavailability [280,281]. Recently, lipid-based nanocarriers and biomimetic magnetic nanoparticles functionalized fourth-gen ABC efflux pump inhibitors have shown pharmacological promise against drug resistant cancers [281,282]. Small interfering RNAs (siRNA) as inhibition for ABC drug efflux pumps were also tested in patients [283,284]. A boost in RNA interference (RNAi) based cancer bioenergetics modulation was observed using nanoplatforms as targeting systems [285,286]. Doxorubicin packed in polyelectrolyte nanoliposomes with Pyruvate kinase muscle isozyme-M2 (PKM2) siRNA reduced oxidative glycolysis, inhibited drug efflux pumps, induced ROS generation, and apoptotic cancer cell death [287]. Additionally, numerous protein tyrosine kinase inhibitors (TKIs) have shown clinical efficacy and hence were authorized by the US Food and Drug Administration (FDA) as cancer therapy [288]. Several clinical trials were conducted using the TKI with conventional cancer drugs [233,234]. However, in clinical settings, most drugs and therapeutic modalities were intercepted with poor bioavailability, low solubility, toxicity, and adverse side effects [289,290,291]. Nanoencapsulation of TKI using lipid base nanoparticles, liposomes, nanopolymers, dendrimers, liposomes, magnetic or silica nanoparticles can provide a plausible targeting approach.

**Figure 4:**
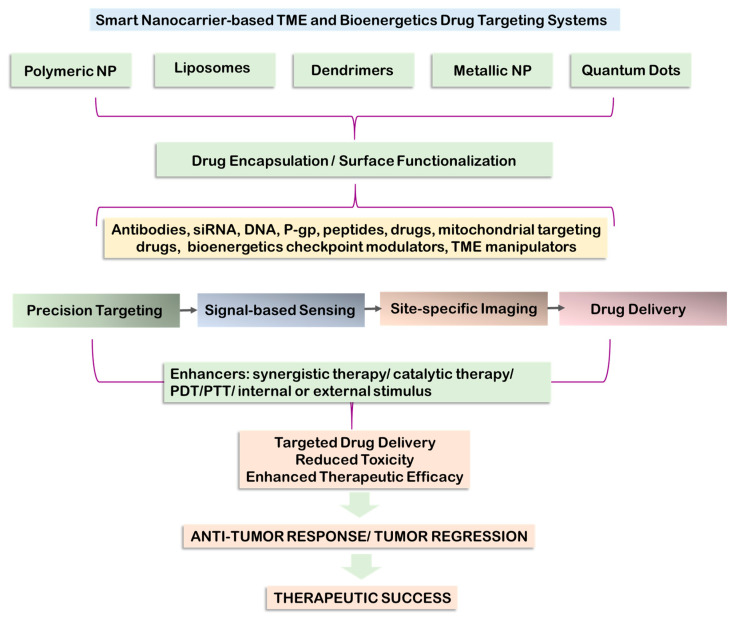
Smart nanocarriers based TME and bioenergetics modulation. Small molecular weight nanoparticles are targeted to metabolic pathways including lactic acid, kynurenine, prostaglandin E2, ROS pathways, and other metabolic targets. Nanoparticles functionalized or encapsulated with drugs demonstrate precise targeting to the destined location due to their small size and physiochemical properties. This advanced technology has enabled the integration of nano-based drug targeting systems with imaging modalities to follow the chemotherapeutics and diagnostic track. Upon localization to the precise location, the drug effect is initiated to induce desired response with reduced toxicity.

An amalgamation of nanomaterials with cancer chemotherapy has presented an amicable solution to overcome chemotherapeutic bottlenecks. These 1–100 nm particles possess unique magnetic, electrical, and optical properties that are extensively harnessed to customize for cancer theranostics. Nanomaterials thus far used in cancer therapeutics span several types to target tumor cells, TME, and the immune system. These particles are modifiable systems that help to overcome toxicity, enhance drug delivery and specificity, and improve bioavailability. Many studies affirm that nanotechnology-based chemotherapeutic approaches that precisely target the mitochondrial function may provide a foothold in increasing the survival rate of patients (Figure 4). Several anti-tumor agents are target mitochondria through nanoparticle-based delivery systems. Mitochondrial intervention through nano-encapsulated or functionalized drugs that lead to the release of cytochrome C has shown promising results. The released cytochrome C facilitates caspase-9 activation through Apaf1, forming an apoptosome [237]. Targeting mitochondrial pathways is instrumental in explicitly inducing cancer cell death without affecting normal cells and eradicating drug resistance [238]. Hybrid nanostructure combinations selectively induced by light/heat (photodynamic/photothermal) are some enhanced nanoplatforms that can be activated after reaching intramitochondrial locations [238,239,240]. Further modifications to photodynamic (PDT) and photothermal (PTT) therapeutic strategies are enhanced by the use of BIODPY dyes. BIODPY-centered theranostics are currently among the most versatile platforms with modifiable functionality and precise cancer cell targetability. BIODPY presents as one of the finest modalities to synergize with the current state-of-art chemotherapeutic drugs to enhance treatment potency and minimize adverse side effects [238]. Metal-based nanoparticles are also tested in mitochondrial drug targeting. Selenium-based functionalized nanomodulators have helped the efficacy of 5-fluorouracil drugs. Encapsulated in poly (D, L-lactide-co-glycolide) nanoparticles, the targeted drug showed enhanced glucose uptake, cytotoxicity, and apoptosis in almost all breast cancer and colon cancer cell lines. A rebalancing or redox status was observed as the central mechanism, while nanoencapsulation increased the sensitivity of the drug [241].

Mitochondrial bioenergetics targeting nanosystems have gained research and therapeutic momentum, but there remains a gap between research and clinical translation. The number of FDA-approved nanodrugs has remained the same over the years. However, upcoming research efforts are visible. It is therefore required to channel research efforts towards an improved clinical translation, wherein the enhanced permeability and retention effects of the nanodrug system are standardized in addition to reducing the generation of protein corona shielding effect.

### 2.7. Conclusions and Insights

Mitochondrial bioenergetics modulations are central to inducing tumor initiation, subsequent progression, and metastasis. A better insight into the mitochondria-centered metabolic reprogramming of cancers is inherent in developing modular treatment opportunities to target critical bottlenecks in tumors, TME, CSC, and CTC, and metastasis. Mitochondria are central to all cancer effector mechanisms that span pH perturbations, redox alterations, cellular phenotype switching, and energy-metabolic skewing towards preferred substrates. The unique role of mitochondria in imparting tumor resistance to the microenvironment also projects them as the perfect therapeutic target. Mitochondria-based selective delivery of nanoformulated chemotherapeutics is a smart methodology for selective, targeted, and safer cancer therapeutics. Nanoparticles have been tested as safe delivery vehicles for cancer targeting, with reduced side effects and concomitant enhancement in drug delivery and therapeutic efficacy. Several therapeutic combinations, such as PDT and PTT, have enhanced the therapeutic success of mitochondria-targeted nanosystems.

### 2.8. Challenges and Future Prospects

Resistant cancer niches, portrayed majorly by CSC, are amongst the most refractory targets of cancer chemotherapy. Being the initiators of tumor occurrence, metastasis, chemotherapeutic drug resistance, and post-treatment recurrence, the eradication of CSC is central to successful cancer treatment. However, the current understanding of CSC is still preliminary with numerous technical lacunae and knowledge gaps. CSCs are physiologically similar to normal stem cells with inherent self-renewal ability, pluripotency, signaling events, and cell surface markers, leaving us with an important research question of how to kill CSC selectively and effectively without affecting the normal stem cells. This requires a thorough understanding of the biochemical and regulatory mechanisms of CSC, which is currently lacking. Needless to mention that the present advancement in the CSC research somewhat levies on the theoretical juncture and requires a practical approach to explore successful therapeutic targets. Current CSC-directed cancer treatment approaches have shown promise, but an insightful understanding of the heterogeneity of CSC niches will profoundly enhance the therapeutic approaches and targeting modalities. Cancer theranostics has seen major improvements with the advent of nanomaterials and, subsequently, smart nanomaterials. It is now amiable to target remote and resistant niches for diagnostic imaging and therapy. In many ways, functionalized or encapsulated nanomaterials have enhanced drug solubility, sustained and targeted release, tracking, and biocompatibility with lesser side effects. Even with the persisting knowledge gaps in the biological intricacies of various TME niches and tumor bioenergetics, the search for the most suitable nanoplatforms for chemotherapeutic targeting and diagnostics remains less directional and arbitrary. It is, therefore, imperative to focus on bridging the understanding of the TME, its immune microenvironment, resistant niches, and factors instrumental in dampening the chemotherapeutic responses. A concerted effort towards eliminating the logical lacunae will also provide answers to the difference in patient response against numerous chemotherapeutic agents.

Though nanomaterials have paved the way forward for chemotherapeutic, diagnostic, and prognostic improvements against various cancers, their clinical translation still needs to be improved. The inherent nature of smart nanosystems can lead to either beneficial or detrimental effects depending on the cellular environment. It must be understood that for a successful nanosystem-based therapeutic regime, the resultant effect must be localized to the cancerous tissues. In addition, technical advancement to improve the target linking efficiency with nanomaterials, its stability, the activity of the linked molecule, and the metabolic mechanism remain to be addressed. Numerous reports of nanomaterial-based toxicity have emerged due to increased oxidative stress, tissue accumulation, inflammation, etc. There are no reports on the ability of nanomaterials to be safely metabolized within the body. This mandates a proper toxicological evaluation and in vitro testing before the nanosystems-chemotherapeutics can be effectively translated into clinical settings. It may be beneficial to establish a methodology and standard for toxicological analyses of nanosystems. Finally, many smart nanosystems have been developed and attained preclinical success. The clinical translation, however, requires a foothold on the thorough understanding of their interaction with the TME and tumor cells. Nonetheless, the impact of modular nanosystems on cancer theranostics leads to the hypothesis that nanosystem-based targeted therapy has profound positive implications.

## Data Availability

Not applicable.
